# Maternal Obesity and Risk of Low Birth Weight, Fetal Growth Restriction, and Macrosomia: Multiple Analyses

**DOI:** 10.3390/nu13041213

**Published:** 2021-04-07

**Authors:** Małgorzata Lewandowska

**Affiliations:** 1 Medical Faculty, Lazarski University, 02-662 Warsaw, Poland; mal2015lewandowska@gmail.com; 2 Division of Gynecological Surgery, University Hospital, 33 Polna Str., 60-535 Poznan, Poland

**Keywords:** maternal obesity, fetal growth, macrosomia, birth weight, weight gain, pregnancy

## Abstract

The associations between maternal pre-pregnancy obesity and low birth weight (LBW, <2500 g) remain inconclusive. Therefore, birth weight in a Polish prospective cohort of 912 mothers was investigated depending on the pre-pregnancy body mass index (BMI). The whole cohort and the subgroup of gestational weight gain (GWG) in the range of the Institute of Medicine (IOM) recommendations, as well as ‘healthy’ women (who did not develop diabetes or hypertension in this pregnancy) were investigated. Adjusted odds ratios (AOR) of the newborn outcomes (with 95% confidence intervals, CI) for obesity (BMI ≥ 30 kg/m^2^) vs. normal BMI (18.5−24.9 kg/m^2^) were calculated using multiple logistic regression. Risk profiles (in the Lowess method) were presented for BMI values (kg/m^2^) and threshold BMI values were calculated. (1) In the cohort, LBW affected 6.6% of pregnancies, fetal growth restriction (FGR) 2.3%, and macrosomia 10.6%. (2) The adjusted risk of macrosomia was more than three-fold higher for obesity compared to normal BMI in the whole cohort (AOR = 3.21 (1.69−6.1), *p* < 0.001) and the result was maintained in the subgroups. A 17-fold higher adjusted LBW risk for obesity was found (AOR = 17.42 (1.5−202.6), *p* = 0.022), but only in the normal GWG subgroup. The FGR risk profile was U-shaped: in the entire cohort, the risk was more than three times higher for obesity (AOR = 3.12 (1.02−9.54), *p* = 0.045) and underweight (AOR = 3.84 (1.13−13.0), *p* = 0.031). (3) The risk profiles showed that the highest BMI values were found to be associated with a higher risk of these three newborn outcomes and the threshold BMI was 23.7 kg/m^2^ for macrosomia, 26.2 kg/m^2^ for LBW, and 31.8 kg/m^2^ for FGR. These results confirm the multidirectional effects of obesity on fetal growth (low birth weight, fetal growth restriction, and macrosomia). The results for LBW were heavily masked by the effects of abnormal gestational weight gain.

## 1. Introduction

The obesity epidemic is a serious public health problem [1]. Importantly, the prevalence of obesity has doubled in the last decade, and this condition affects, on average, a third of women in reproductive age [2,3]. Maternal obesity is a known modifiable risk factor for adverse outcomes in pregnancy [4,5,6,7]. However, not all obesity effects have been unequivocally established, including its association with low birth weight (LBW) [8,9], defined according to World Health Organization (WHO) as birth weight <2500 g regardless of gestational age [10]. 

Both excessive and too low birth weight carries many adverse health consequences, both short-term and long-term, which determines a worse transition of a child into adulthood [1,11]. Therefore, establishing the role of maternal obesity (the modifiable risk factor) is a prerequisite for implementing the most beneficial interventions to promote fetal/child health [1]. 

Macrosomic neonates have a significantly higher risk of childhood obesity (by 50%) and a higher risk of premature cardiovascular death (by 35%) [1,12,13,14,15]. This intergenerational cycle of obesity fits into the theories of fetal development non-infectious diseases in line with the concept of Developmental Origins of Health and Disease (DOHaD) [1]. Macrosomia has also been linked to neuro-developmental disorders in children [3]. Many studies have shown associations of maternal obesity with macrosomia risk [12,13,14,15].

The effects of low birth weight (LBW, <2500 g) are also serious and are associated with higher infant mortality [10]. In addition to neuropsychiatric disorders and reduced educational achievement [3,11], and stunting and underweight in offspring [16], LBW newborns may also have a higher risk of obesity, diabetes and cardiovascular disease in adulthood [3,11]. 

The studies conducted so far show divergent results of the analyses of the relationships between maternal obesity and LBW risk. Several studies have found an effect of maternal obesity on a higher risk of LBW [17,18], but other studies have not confirmed this [8,9,19]. Low birth weight (LBW) has rather been associated with maternal low body mass index (BMI) or low mid-upper arm circumference (MUAC) [16,20,21,22], as well as other factors (e.g., smoking or maternal hypertension) [23,24]. However, the associations between maternal obesity and a higher risk of fetal growth restriction (defined as the inability of the fetus to reach its full growth potential) were found [25]. Importantly, not all analyses investigated the effects of pre-pregnancy obesity on birth weight after excluding the effects of gestational weight gain or other confounding factors. 

The main aim of this study was to evaluate the relationship between pre-pregnancy obesity and the risk of low birth weight (LBW), fetal growth restriction (FGR) and macrosomia, applying multiple statistical methods and in a prospectively pooled cohort. Additional goals were as follows: (1) Assessing different BMI categories, as well as evaluating BMI as a continuous variable and determining a BMI threshold for each outcome; and (2) evaluating the entire cohort as well as the subgroup of women with normal gestational weight gain (GWG) and the ‘healthy’ women’s subgroup (women who did not develop diabetes or hypertension during this pregnancy).

## 2. Materials and Methods

This study was conducted at the Obstetrics and Gynecology Hospital of the Poznan University of Medical Sciences (Poznań, Poland); it is a medical facility of the highest (third) degree of reference for obstetrics and neonatology in the region, and the number of deliveries in this hospital amounts to 6000–8000 per year. Recruitment to the original cohort was carried out in 2015−2016.

### 2.1. Ethical Statement

All recruitment and research procedures were carried out in accordance with the Helsinki Declaration. Participants volunteered to participate in the study and signed their informed consent to participate before completing the questionnaires. All procedures of this study were approved by the Bioethics Committee of the University of Medical Sciences, Poznań, Poland (under number 769/15).

### 2.2. Method and Data Collection

This was a prospective cohort study. The primary cohort was recruited at the end of the first trimester of pregnancy (according to the agreed inclusion criteria). Subsequently, the pregnancy results were taken from medical records.

The inclusion criteria for this study were as follows: Caucasian race, residence in the Wielkopolska region, 10−14th week of gestation (during recruitment) and singleton pregnancy, mother’s age 18−45 years (at the time of conception), as well as lack of any chronic diseases (except abnormal pre-pregnancy weight), in particular the lack of pre-existing hypertension or diabetes mellitus, immunological disorders and inflammatory diseases, liver diseases and kidney diseases, coagulation disorders or neurological disorders. Both primiparous and multiparous women were included in the study. Another inclusion criterion was the absence of fetal defects and the delivery of a phenotypically normal child with a gestational age greater than or equal to 25 weeks.

Recruitment was carried out at the Central Laboratory of the research center where women reported for standard laboratory tests during pregnancy. Information about the recruitment for this scientific study was posted in the Laboratory, in a very visible place. Women reported their willingness to participate in this study independently and voluntarily.

During recruitment (carried out on the 10–14th week of gestation), personal questionnaires were used to collect preliminary data (including data on body weight before pregnancy). Information on the current pregnancy and its course to date, demographic and socio-economic data, as well as information on obstetric and gynecological history, information on pre-pregnancy and pregnancy medications (including taking folic acid or other multivitamin supplements/multi-micronutrient preparations) was collected. Data on the use of stimulants, and smoking and alcohol consumption was gathered, as well as information on the history of diseases in the family. The participants completed the questionnaire (independently) in the presence of a midwife. 

After the childbirth, followed the second stage of data collection. Information on the results of the newborn and maternal health, including maternal/prenatal weight, was taken from the medical records. Another questionnaire allowed for the collection of additional data from participants (they were contacted by phone or e-mail after the 12th week of puerperium): it included the information on the course of the puerperium, arterial pressure profile in the puerperium, and the information on possible changes in smoking habits or using other stimulants during pregnancy (all the participants declared refraining from alcohol consumption during pregnancy).

The overall flow pattern in this study was as follows: (i) 1300 women (eligible for inclusion at 10–14 week of gestation) volunteered to participate in this study over a 12-month period. All these women were invited to take part in the study and completed the questionnaires. (ii) After delivery (and collection of pregnancy outcomes data), 388 mothers were excluded from the study for the following reasons: Delivery <25th week of gestation, childbirth with congenital diseases, cases of severe infections or thromboembolism during pregnancy, arterial hypertension diagnosed before the 20th week and/or diabetes mellitus diagnosed before the 18th week, as well as the lack of cooperation (*n* = 48) and missing data (*n* = 340). Finally, 912 mothers were qualified for analyses.

The main aim of this study was to evaluate the relationship between pre-pregnancy obesity and the risk of macrosomia or low birth weight (LBW). The minimum sample size (for each analysis) was calculated using the formula for a single proportion:

(1) $$n=Z_{\frac{\alpha }{2}\;\;}^{2}\frac{p\cdot \left(1-p\right)}{d^{2}}$$

(for 95% confidence intervals and *α* = 5%. ‘*Z*’: critical value of normal distribution at *α*/2 and *Z*-value = 1.962; ‘*d*’: margin of error; ‘*p*’: sample proportion). 

The minimum sample size was 864 for an error value of *d* = 0.02 (2%) and for the macrosomia proportion *p* = 0.1 (10%), i.e., the mean frequency cited in the literature [26,27]. The minimum sample size was 625 for the error value of *d* = 0.02 (2%) and for the low birth weight (LBW) proportion *p* = 0.07 (7%), cited in the literature [11]. Our sample size (*N* = 912) was good enough to discover the relationships of interest to us.

In the cohort presented (*N* = 912), 97 (10.6%) women gave birth to newborns with macrosomia >4000 g, 60 (6.6%) women had children with low birth weight (LBW) <2500 g, 755 women (82.8%) had newborns between 2500−4000 g and 21 (2.3%) women had a child with fetal growth restriction (FGR). Other outcomes were as follows: 146 (16%) participants developed gestational diabetes mellitus (GDM) (21 cases treated with insulin (GDM-2)); 137 (15%) participants developed pregnancy-induced hypertension (24 preeclampsia (PE) cases); 65 (7.1%) participants gave birth before 37th week; 382 (41.9%) participants had a cesarian section.

### 2.3. Definitions of Independent Variables

The main independent variable studied was the pre-pregnancy body mass index (BMI). BMI was calculated as the quotient of pre-pregnancy weight in kg (self-reported) and maternal height squared (meter^2^) (from medical reports). In this analysis, BMI was assessed in the four following categories (according to World Health Organization definitions): (i) Underweight (<18.5 kg/m^2^); (ii) normal weight/BMI (18.5–24.9 kg/m^2^); (iii) overweight (25.0–29.9 kg/m^2^); and (iv) obesity (≥30 kg/m^2^). The reference category was ‘normal BMI’. BMI was also assessed as a continuous variable (kg/m^2^).

The second independent variable under investigation was gestational weight gain (GWG). GWG was the difference between the weight before childbirth (from medical reports) and the weight before pregnancy (self-reported). The GWG was assessed for each pre-pregnancy BMI category, and the normal GWG ranges (as defined by the Institute of Medicine (IOM) in 2009) were as follows: (i) 12.5–18 kg for underweight; (ii) 11.5–16 kg for normal BMI; (iii) 7–11.5 kg for overweight; and (iv) 5–9 kg for obesity. In this analysis, GWG was assessed in the three following categories (regardless of pre-pregnancy BMI): (i) GWG above the range; (ii) GWG in the range; and (iii) GWG below the range. The reference category was ‘GWG in the range’.

### 2.4. Definitions of Dependent Variables

The dependent variables studied were the categories of abnormal birth weight. The weight of the newborns (in grams) was measured immediately after delivery using an automatic scale. The definitions of birth weight in grams were as follows: macrosomia is >4000 g regardless of gestational age [12]; low birth weight (LBW) is <2500 g regardless of gestational age [10]; the correct birth weight is 2500−4000 g (which was a reference category in statistical analyses). Birth weight expressed in percentiles (for a specific population, fetal sex and gestational age) covered large-for-gestational age (LGA) (the birth weight >90th percentile), appropriate-for-gestational age (AGA) (the weight 10–90th percentile) and small-for-gestational age (SGA) (the birth weight <10th percentile without cases of fetal growth restriction), according to the Polish percentile grids as described in our earlier work [13]. SGA refers to fetuses that are constitutionally small, consistent with their (genetic) growth potential [28]. 

Fetal growth restriction (FGR) was defined as the inability of the fetus to reach its full (genetic) growth potential [10,28] and was diagnosed by ultrasound during pregnancy. In this study, the detailed diagnostic criteria for each case are not known (were not available) and may have been different in tests performed by different physicians [29]. The ultrasound examination included determination of gestational age, series of biometric measurements (to establish growth potential), and evaluation of the umbilical artery. The medical records found 21 newborns diagnosed with FGR, including 16 newborns with a birth weight <10th percentile, and five newborns with a birthweight ≥10th percentile.

The gestational age rating was based on ultrasound examination (crown-rump length (CRL) was assessed between the 10th and 13th (+6 days) week).

### 2.5. Covariates

The following covariates were included in this study (Table 1): Gestational weight gain (GWG), primiparity, maternal age, maternal height, smoking in the first trimester, fetal sex, preeclampsia and gestational diabetes mellitus in this pregnancy, and gestational age at childbirth, as well as prior pregnancy-induced hypertension [24,28,30,31]. Among smoking categories, ‘First trimester smoking’ was used as a covariate because it was associated with a statistically significantly higher risk of LBW (and FGR) and the result was stronger than for ‘smoking before pregnancy’, according to our previous analysis [23].

### 2.6. Statistical Analyses

Statistica software, version 13, was used for the analyses (TIBCO, Palo Alto, CA, USA). Maternal characteristics were compared between mothers with obesity (BMI ≥ 30 kg/m^2^) and mothers with normal BMI (18.5−24.9 kg/m^2^), and were described using median (and interquartile ranges, IQR) or by the number (and percentage). 

The Mann–Whitney U test was used for comparisons of continuous variables (the variables were not normally distributed) between women with obesity and normal BMI; the assessment of the normality of the data distribution was performed using the Shapiro–Wilk test. For comparisons of categorical ordered categories, the Cochran–Armitage test for trend was calculated, and for binomial categories the Pearson chi-square test (or Fisher exact test when Cochran assumption was not met) was used. The *p*-value < 0.05 was assumed to be significant.

To achieve the goal of the study, raw odds ratios (OR and 95% confidence intervals (CI) of each newborn outcome for categories of pre-pregnancy BMI were calculated in a univariate logistic regression (normal BMI was a reference category). Adjusted odds ratios (AOR) (and 95% CI) were calculated using multiple logistic regression after being adjusted to covariates listed in the section above. The statistical significance (*p*-value) of OR/AOR was determined by Wald’s test. A *p*-value < 0.05 was assumed to be significant.

Causal mediation analysis (mediation effect of GWG) was also performed; if, after adding a variable (GWG) to the logistic regression model, the relationship between the dependent variable (newborn outcome) and the independent variable (obesity) decreases or completely disappears, mediation effect of the GWG can be calculated. ACME coefficient (with 95% confidence intervals) describes to us the effect of mediation (ACME: Average causal mediation effects); if it is statistically significantly different from 0 then there is a mediation effect. A *p*-value < 0.05 was assumed to be significant.

These analyses were performed for the entire cohort as well as the subgroup of women with normal gestational weight gain (GWG) (in the range of the Institute of Medicine (IOM) recommendations) and the ‘healthy’ women’s subgroup (women who did not develop diabetes or hypertension during this pregnancy).

Graphs were also created showing the risk profiles of each newborn outcome subject for the BMI as a continuous variable (OR and 95% confidence intervals). The risk profiles were created using the Lowess method. We calculated the BMI threshold value (for an OR = 1) above (or below) which the odds ratios of low or excessive birth weight were increasing.

## 3. Results

### 3.1. General Characteristics of the Cohort and Obese Mothers

In the cohort, 593 (65%) women had normal BMI (18.5−24.9 kg/m^2^), 5.2% had underweight (BMI < 18.0 kg/m^2^), 19% had overweight (25−29.9 kg/m^2^), 10.8% had obesity (BMI ≥ 30 kg/m^2^), while four women (0.44%) had grade III obesity (BMI ≥ 40 kg/m^2^), 338 (37.1%) participants had gestational weight gain (GWG) in the range of the Institute of Medicine (IOM) recommendations, and 36.8% of the mothers had GWG above the recommendations. 

Table 2 presents the characteristics of the obese women (vs. women with normal BMI). The median pre-pregnancy BMI in the group of the obese women was 32.7 (kg/m^2^) (25–75%: 31.1−35.3) and the median pre-pregnancy weight was 90 kg (25–75%: 87−97). Compared to the women with normal BMI, the obese women had a statistically significantly higher percentage of gestational weight gain (GWG) above the range (55.1% vs. 28.7%), although the median GWG in the group of the obese women was significantly lower (some of the obese women had a very high weight loss during pregnancy). The obese women were statistically significantly older, as well as comprised a higher proportion of smokers and women with lower financial status or lower education. A percentage of women developing preeclampsia (PE) or gestational diabetes mellitus (GDM) during the current pregnancy was also higher (Table 2).

In the group of mothers with obesity (compared to the women with normal BMI) was found a higher percentage of macrosomia >4000 g (23.5% vs. 7.4%) as well as a higher percentage of low birth weight (LBW) (<2500 g) (13.3% vs. 5.4%) and fetal growth restriction (FGR) cases (5.2% vs. 2.0%).

### 3.2. Maternal Obesity and Macrosomia Risk

Table 3 presents the crude (OR) and adjusted odds ratios (AOR) of macrosomia (>4000 g) for pre-pregnancy BMI categories, calculated in the entire cohort as well as in the subgroups. The adjusted odds ratios (AOR, with 95% confidence intervals (CI) were calculated using multiple logistic regression. 

In the entire cohort, maternal obesity (vs. normal BMI) was associated with more than three-fold higher adjusted risk of macrosomia (AOR = 3.21 (1.69−6.1), *p* < 0.001) and the result was maintained in the subgroup of ‘healthy’ women, i.e., women who did not develop either diabetes or hypertension in the current pregnancy (AOR = 4.33 (1.71−10.96), *p* = 0.002), as well as in the subgroup of GWG in the range (AOR = 3.94 (1.2−12.95), *p* = 0.024). 

In the risk of large-for-gestational age (LGA), similar results for maternal obesity were obtained in the entire cohort and subgroup of ‘healthy’ women. However, these results did not persist in the subgroup of GWG in the range.

**Figure 1:**
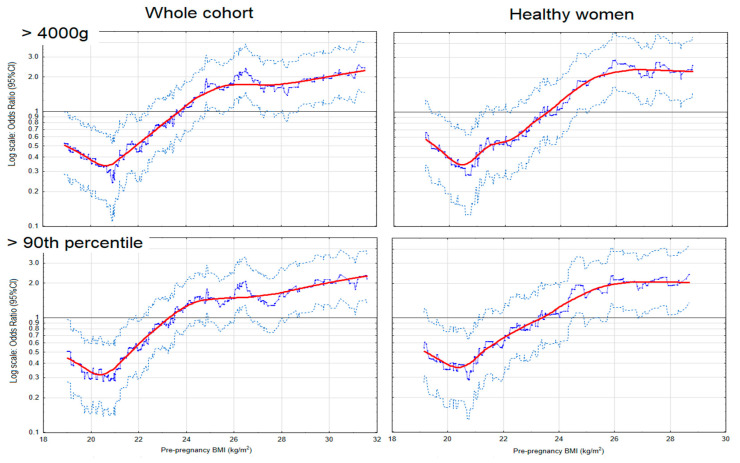
The risk profiles of excessive birth weight for pre-pregnancy body mass index (BMI), in the whole cohort and subgroup of ‘heathy’ women (women who did not develop either hypertension or diabetes in the current pregnancy). The graphs illustrate the changes in the odds ratios (OR) of the birth weight for the changes in the BMI values (kg/m^2^). The odds ratios (blue points) were calculated on a sliding window (for 100 observations) and the red curves show the risk profiles (smoothed with the Lowess method). The points above the horizontal line indicate an increased risk, and the points below this line correspond to a reduction in risk. The light blue points represent the upper and lower limits of the 95% confidence intervals (CI) for OR.

Figure 1 presents the risk profiles of macrosomia (>4000 g) for pre-pregnancy BMI as a continuous variable (kg/m^2^) in the entire cohort and ‘healthy’ subgroup. The risk graphs estimated in the entire cohort show that higher BMI before pregnancy was as-sociated with a marked increase in the crude odds ratios (OR) of macrosomia and LGA. The threshold BMI above which the macrosomia and LGA risk increased was 23.7 and 23.2 kg/m^2^ (respectively). The relationships of excessive BMI with the macrosomia and LGA was maintained in the subgroup of ‘healthy’ women (Figure 1) and normal GWG (Figure S1). The threshold BMI values were similar in the subgroups and whole cohort. 

Odds ratios for extreme BMI values are not shown because the measurement ‘sliding window’ covers 100 cases (and the tested BMI value falls in the middle of this window). 

### 3.3. Maternal Obesity, and Low Birth Weight (LBW) and Fetal Growth Restriction (FGR)

**Figure 2:**
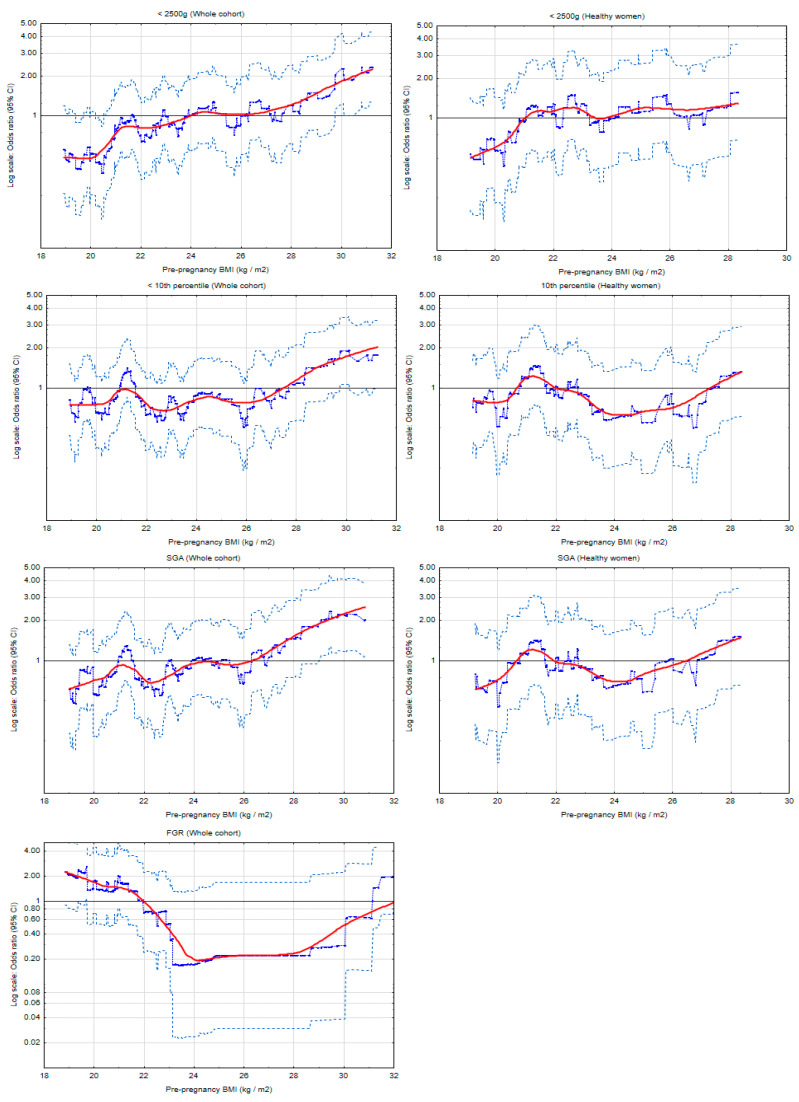
The risk profiles of lower birth weight for pre-pregnancy body mass index (BMI), in the whole cohort and subgroup of ‘heathy’ women (women who did not develop either hypertension or diabetes in the current pregnancy). The graphs illustrate the changes in the odds ratios (OR) of the birth weight for the changes in the BMI values (kg/m^2^). The odds ratios (blue points) were calculated on a sliding window (for 100 observations) and the red curves show the risk profiles (smoothed with the Lowess method). The points above the horizontal line indicate an increased risk, and the points below this line correspond to a reduction in risk. The light blue points represent the upper and lower limits of the 95% confidence intervals (CI) for OR. SGA: Small-for-gestational age (birth weight < 10th percentile without FGR cases); FGR: Fetal growth restriction.

Figure 2 and Figure S1 present the risk profiles of LBW (<2500 g), FGR and small-for gestational age (SGA) for pre-pregnancy BMI as a continuous variable (kg/m^2^) in the entire cohort and subgroups, calculated on a sliding window for 100 (Figure 2) or 30 (Figure S1) observations.

Figure 2 shows that in the entire cohort, the highest BMI was associated with the highest odds ratios of LBW and SGA, and the threshold BMI was 26.2 kg/m^2^ and 26.2 kg/m^2^, respectively. The associations were weaker in the subgroup of ‘healthy’ women (women without gestational diabetes mellitus or hypertension in the current pregnancy). Importantly, the threshold BMI values differed in the subgroups. Figure S1 shows that in the subgroup of normal GWG, the results for LBW and SGA were similar but the threshold BMI were different (especially for LBW) (the risk profiles were calculated on a sliding window for 30 observations. 

The FGR risk profile was U-shaped (Figure 2 and Figure S1): Underweight and obesity were associated with a higher FGR risk. The threshold BMI values were 22.0 kg/m^2^ and 31.8 kg/m^2^, and BMI between 24−28 kg/m^2^ was associated with the lowest risk of FGR (Figure 2). The number of FGR cases in the subgroups decreased significantly (*n* = 11) and, therefore, it was not possible to build the graph.

Table 4 presents the crude (OR) and adjusted odds ratios (AOR) of low birth weight (LBW, <2500 g) for pre-pregnancy BMI categories, calculated in the entire cohort as well as in the subgroups. The adjusted odds ratios (AOR, with 95% confidence intervals (CI)) were calculated using multiple logistic regression. 

In the entire cohort, maternal obesity (vs. normal BMI) was associated with more than three-fold higher raw LBW risk (OR = 3.39 (1.69−6.81), *p* = 0.001), but the result was not maintained in multiple analyses or in the subgroup of ‘healthy’ women.

Importantly, in the subgroup of GWG in the range, a 17-fold higher adjusted LBW risk for maternal obesity was found (AOR = 17.42 (1.5−202.6), *p* = 0.022). In this subgroup, overweight was also associated with a higher adjusted risk of LBW. However, the relationship between underweight and LBW did not persist in the multiple model. 

No statistically significant relationship was found between obesity and small-for-gestational-age (SGA) (birth weight <10th percentile without fetal growth restriction cases).

Several significant OR scores became insignificant after adjustment (AOR). The mediation effect of the GWG was tested (Table S1) and this effect was found.

Table 5 presents the crude odds ratios (OR) and adjusted odds ratios (AOR) of fetal growth restriction (FGR) for pre-pregnancy BMI categories, calculated in the entire cohort as well as in the subgroups. The adjusted odds ratios (AOR, with 95% confidence intervals (CI)) were calculated using multiple logistic regression. 

In the entire cohort and subgroups, the relationship between FGR and BMI was U-shaped (or inverted ‘J’). In the entire cohort, the adjusted FGR risk was more than three times higher for maternal obesity (AOR = 3.12 (1.02−9.54), *p* = 0.045) and underweight (AOR = 3.84 (1.13−13.0), *p* = 0.031), compared to normal BMI.

In the subgroup of GWG in the range, underweight women had a 11-fold higher FGR risk (AOR = 11.82 (1.95−71.6), *p* = 0.007) compared to women with normal BMI. Obese women had a four-fold higher adjusted odds ratio of FGR (AOR = 4.06 (0.38−43.07), *p* = 0.245). 

In the subgroup of ‘healthy’ women, the associations were weaker and statistically insignificant.

### 3.4. Effects of Gestational Weight Gain (GWG) on the Newborn’s Weight

Table S2 presents crude odds ratios (OR) and adjusted odds ratios (AOR) of abnormal birth weight for gestational weight gain (GWG) categories, calculated in the subgroup of normal pre-pregnancy BMI, using multiple logistic regression.

In the subgroup of normal BMI, statistically significant associations between GWG above the range and a higher risk of macrosomia (AOR = 2.16 (1.07−4.35), *p* = 0.031) and LGA (AOR = 2.35 (1.18−4.67), *p* = 0.015) were found. 

GWG below the range was associated with a higher risk of LBW (AOR = 3.71 (1.1−12.55), *p* = 0.035) and FGR (AOR = 3.74 (0.97−14.48), *p* = 0.056).

## 4. Discussion

The results of these analyses showed associations of pre-pregnancy obesity with a higher risk of low birth weight (LBW), fetal growth restriction (FGR) and macrosomia. The results were obtained using multiple logistic regression and after excluding effects of abnormal gestational weight gain (GWG) and hypertension, and diabetes mellitus. An analysis of BMI as a continuous variable (risk profiles) confirmed that the highest BMI values were found to be associated with a higher risk of these three newborn outcomes. The relationship between FGR and BMI was U-shaped. This study shows that the risk of excessive or too low birth weight begins to increase starting from BMI values at the upper end of the normal range and upwards (the threshold BMI).

It should be emphasized, that the size of some subgroups in this study was small, (some confidence intervals were quite wide) but the relationships found were statistically significant, which means that the results obtained were not accidental.

The significance of the results obtained has several aspects. Firstly, these results suggest/confirm the multidirectional effects of obesity on fetal growth, confirming the need to investigate the mechanisms of the effects of obesity on placental function and fetal development. Secondly, this study identified strong confounders in investigating the effects of various BMI categories (such as gestational weight gain). The clinical implications of all the results include the need to seek interventions aimed at changing dietary habits and normalizing women’s weight before pregnancy in order to promote fetal health.

Our study has added several results/effects to the literature. The available literature on the associations between maternal obesity and low birth weight (LBW, <2500 g) provides inconsistent results; several studies showed relationships between pre-pregnancy obesity and a higher risk of low birth weight, LBW (<2500 g) [17,18,32], and many studies presented associations of LBW risk with lower maternal weight [16,20,21,22,32]. However (contrary to our analysis) these were retrospective studies, they differed in the size of the studied populations, not all analyses investigated the effects of maternal obesity on birth weight using multiple statistical models and after excluding the effects of gestational weight gain out of the range, hypertension, and diabetes. The risk profiles were not assessed in any study. Many previous studies confirmed the associations between a higher macrosomia risk and maternal obesity [13,14,15,17,18,20] or between fetal growth restriction (FGR) risk and maternal obesity [3,18,25,33] or underweight [33,34,35]. However, these studies were retrospective and also did not include subgroup analyses or risk profiles (as was the case in our study).

This study included women with singleton pregnancy who were recruited prospectively at the end of the first trimester, and one of the inclusion criteria was the absence of chronic disease (including diabetes and hypertension). The results obtained in this study were adjusted for gestational weight gain (GWG), primiparity, maternal age, maternal height, smoking in the first trimester, fetal sex, diabetes, and hypertension in this pregnancy, gestational age at childbirth, and hypertension in previous pregnancy [24,28,30,31]. In this study, the subgroups of normal gestational weight gain (GWG) and ‘healthy’ women were investigated separately. The risk profiles made it possible to determine (and compare) BMI threshold values in the development of excessive and too low birth weight.

In this cohort, the percentage of macrosomic neonates (10.6%) was higher than the percentage of LBW neonates (6.6%), which is consistent with the literature [11,26,27]. The number of fetal growth restriction (FGR) cases was low (2.3% of pregnancies) (Table 2), but this was a low-risk cohort. In this study, 29.7% of women had overweight and obesity, and obese women had a statistically significantly higher percentage of excessive gestational weight gain (GWG) compared to the women with normal BMI (55.1% vs. 28.7%, *p* < 0.0001) (Table 2).

This study showed that the analysis based on BMI categories may have significant limitations. The low birth weight (LBW) and small-for-gestational age (SGA) risk profiles were similar (Figure 2). However, there were differences between LBW and SGA risk scores obtained from the comparison of the categories (BMI ≥ 30 kg/m^2^ vs. normal BMI) (Table 4); the results for SGA were weaker. It may be due to different BMI thresholds for different newborn outcomes (Figure S1). Importantly, these outcomes also have different definitions: LBW was defined as birth weight <2500 g regardless of gestational age; SGA was defined as birth weight <10th percentile (for gestational age and fetal sex, for a specific population) without cases of fetal growth restriction.

This analysis showed that abnormal gestational weight gain (GWG) has a stronger confounding effect on the relationship between maternal obesity and birth weight than might be expected. A 17-fold higher adjusted low birth weight (LBW) risk for maternal obesity was found in the subgroup of normal GWG, but not in the entire cohort (Table 4). At the same time, insufficient GWG (compared to normal GWG) increased the risk of LBW by three times (Table S1). As the GWG is considered a major confounding factor, the mediation effect of the GWG was tested and this effect was found (Table S1). Additionally, there was a difference between the threshold BMI for LBW in the whole cohort (it was higher) and in the subgroup of normal GWG (where it was lower) (Figure S1). 

The results obtained in the ‘healthy’ women’s subgroup (Figure 2 and Table 4) showed weaker associations of BMI with the risk of LBW and SGA compared to the results in the entire cohort, which suggests the influence of pregnancy-induced hypertension (PIH) and/or gestational diabetes mellitus (GDM) on the effects studied.

The mechanisms of the relationships between maternal obesity and too low or excessive birth weight of a newborn are not fully understood. Placental functions, maternal nutritional status and the flow of nutrients from the mother to the fetus are considered to be the main determinants of fetal growth, in addition to genetic factors [36,37,38]. Placental abnormalities and other factors (e.g., maternal disease) reduce the growth of the fetus by impairing placental functions and are associated with the development of low birth weight (LBW) and fetal growth restriction (FGR) [3,28]. 

Disorders accompanying obesity, such as chronic inflammation, oxidative stress, insulin resistance and dysregulation of neurohormones and cytokines as well as epigenetic changes affect the function of the placenta and its ability to transport of nutrients [39,40]. Among many processes in obese mothers, opposing regulatory roles of the two processes were found [3]. (1) It was found that higher levels of maternal TNF-α, IL-6 interleukin, insulin, and leptin may be associated with a higher risk of fetal hypertrophy (these markers are associated with stimulate the activity of nutrient transporters in the placenta) [3]. (2) On the other hand, when the maternal obesity was accompanied by increased levels of IL-1β interleukin (which inhibits the transport of nutrients induced by insulin stimulation) and by an increase in the level of soluble FMS-like tyrosine kinase (sFLT) with a decrease in the level of placental growth factor (PlGF), markers involved in angiogenesis (which results in a decrease in blood flow through the placenta), there was a decrease in fetus growth [3,41,42].

In randomized intervention studies in pregnant women, a reduction in gestational weight gain (GWG) was achieved, however the improvement in the pregnancy outcomes was poor [1,43]. This suggest that pre-pregnancy obesity/overweight should be the therapeutic target. Interventions should be multi-directional (involving diet, physical activity, and health-promoting behavior) and should be carried out by multidisciplinary teams [1]. Dietary guidelines (worldwide and in Poland) for pregnant women have been described in our previous works [4,13]. 

### Advantages and Limitations

The advantages of this study include the following: assessment of the role of maternal obesity in the prospective cohort (when pregnancy outcomes are unknown during recruitment), the use of multiple statistical analysis (taking into account potential confounding factors), as well as examining a subgroup of mothers with normal pregnancy weight gain and ‘healthy’ women. However, other adjustment models are also possible. The great advantage of this study is the additional plotting of risk profiles for each BMI value and the calculation of the BMI threshold; this allowed to find (and partially explain) the limitations resulting from the examination of the BMI categories. Ours is possibly the first prospective cohort study examining the relationship between maternal obesity/BMI and birth weight in such detail.

This study also has some limitations. One is that pre-pregnancy weight was self-reported, though in fact this is a common practice. The division into subgroups resulted in reducing the sample size (some confidence intervals were quite wide), but statistical significance confirms that these results are not accidental. The specific diagnostic criteria for each case of fetal growth restriction (FGR) are not known (were not available) and may have been different in tests performed by various physicians. All participants reported not using alcohol during pregnancy, which may have been influenced by social requirements; this may affect the results because alcohol consumption is one of the factors that increase the risk of LBW. The number of FGR cases was small and, therefore, the number of confounding factors used was smaller. The low number of FGR cases suggests that the results for this outcome should be treated as pilot study results, although, on the other hand, the relationships found were statistically significant. 

## 5. Conclusions

This multiple analysis performed in a prospectively collected cohort showed that pre-pregnancy obesity increased both the risk of macrosomia and low birth weight (LBW) as well as fetal growth restriction (FGR). The risk profiles showed that the risk of abnormal birth weight already increased for BMI values at the upper limit of the normal BMI range. The obtained results suggest multidirectional effects of obesity on fetal growth and confirm the continuing need to seek interventions in order to normalize the nutritional status of women planning pregnancy to promote children’s health.

## Figures and Tables

**Table 1:** Characteristics of the independent variables and covariates.

Variables	Definitions and Categories	Reference Category/ Covariates	Description
Pre-pregnancy BMI	Defined as the quotient of pre-pregnancy weight (in kg) and height (in meters) squared - was assessed in the four following categories: (1) Underweight (<18.5); (2) normal weight (18.5–24.9); (3) overweight (25.0–29.9); (4) obesity (≥30) - was assessed as a continuous variable	Reference: -normal weight	Self-reported
GWG	Calculated as the difference between the weight before childbirth and the weight before pregnancy - was assessed in the three following categories (according to the 2009 Institute of Medicine recommendations): (1) above the range; (2) in the range; and (3) below the range.	Reference: -GWG in the range	From medical reports
Maternal age	As completed maternal age at conception - was assessed as a continuous variable (years)	The covariate	From medical reports
Primiparity	Parity was assessed in the two following categories: (1) primiparity i.e., zero prior delivery; (2) multiparity (≥1 prior deliveries)	The covariate	From medical reports
Smoking	-was assessed in the three following categories: (1) Women who had never smoked; (2) smokers (women who had smoked before pregnancy); (3) smokers in the first trimester	The covariate: -smokers in the first trimester	Self-reported
Fetal sex	-was assessed in the two following categories: (1) Son; (2) daughter	The covariate	From medical reports
Gestational age	The gestational age rating was based on ultrasound examination (crown-rump length (CRL) was assessed between 10th and 13th (+6 days) week) -was assessed as a continuous variable	The covariate	From medical reports
Gestational diabetes mellitus (GDM)	In order to diagnose GDM, an oral glucose tolerance test (OGTT) for 75g of glucose on empty stomach (2-h test) was performed in 24−28th gestational week.	The covariate	From medical reports
Preeclampsia/ Pregnancy-induced hypertension (PIH)	PIH was defined as blood pressure (systolic and diastolic) ≥ 140/90 mmHg, developing de novo after the 20th gestational week (obtained in at least two measurements four hours apart, and measured with an oscillometric device in a sitting position). Preeclampsia (PE) was diagnosed when this arterial hypertension was accompanied by the following organ disorders (de novo development): renal dysfunction and/or hepatic disorders and/or thrombocytopenia, visual and/or cerebral disorders, or pulmonary edema.	The covariate: -preeclampsia -prior PIH	From medical reports

BMI: body mass index; GWG: gestational weight gain.

**Table 2:** General characteristics of obese women and newborn outcomes.

	Pre-Pregnancy Normal BMI (*n* = 593)	Pre-Pregnancy Obesity (*n* = 98)	
Characteristics	Median (IQR) or *n* (%)	Median (IQR) or *n* (%)	*p* *
Pre−pregnancy weight (kg)	60 (55−65)	90 (87−97)	<0.0001
Pre−pregnancy BMI (kg/m^2^)	21.7 (20.3−23.2)	32.7 (31.1−35.3)	<0.0001
GWG (kg)	14 (11−17)	11 (7−16)	<0.0001
GWG categories			<0.0001
GWG above the range	170 (28.7%)	54 (55.1%)	
GWG in the range	246 (41.5%)	25 (25.5%)	
GWG below the range	177 (29.8%)	19 (19.4%)	
Primiparous women	250 (42.2%)	39 (39.8%)	0.661
Maternal age	35 (30−37)	36 (33−38)	0.004
Smoking	90 (15.2%)	25 (25.5%)	0.011
Education <12 years **	30 (5.8%)	19 (22.4%)	<0.0001
Lower financial status **	58 (19.2%)	30 (50.8%)	<0.0001
Pregnancy outcomes			
Fetal sex, daughter	287 (48.4%)	46 (46.9%)	0.789
Gestational age (weeks)	39 (38−40)	39 (38−40)	0.513
Birth <37th week	37 (6.2%)	15 (15.3%)	0.002
Birth weight (grams)	3390 (3090−3670)	3620 (2960−3980)	0.021
Birth weight categories			<0.0001
<2500g	32 (5.4%)	13 (13.3%)	
2500−4000g	517 (87.2%)	62 (63.3%)	
>4000g	44 (7.4%)	23 (23.5%)	
Birth weight categories			<0.0001
<10th percentile	42 (7.1%)	10 (10.2%)	
10−90th percentile	503 (84.8%)	64 (65.3%)	
>90th percentile	48 (8.1%)	24 (24.5%)	
Fetal growth restriction (FGR)	12 (2.0%)	5 (5.2%)	0.065
Preeclampsia (PE)	9 (1.7%)	9 (13.4%)	<0.0001
GDM	79 (13.3%)	32 (32.7%)	<0.0001
Cesarean section	239 (40.3%)	57 (58.2%)	0.001
APGAR in 5th minute <7	2 (0.3%)	(0%)	1

* The Mann–Whitney U test was used for comparisons of continuous variables (the variables were not normally distributed), for categorical ordered categories Cochran–Armitage test for trend was calculated, and for binomial categories the Pearson chi-square test (or Fisher exact test when Cochran assumption was not met) was used (*p* < 0.05 was assumed to be significant); ** Analyses for available data. BMI: Body mass index; GWG: Gestational weight gain; GDM: Gestational diabetes mellitus; APGAR: Appearance, pulse, grimace, activity, and respiration.

**Table 3:** The adjusted odds ratios of excessive birth weight for pre-pregnancy BMI, in the whole cohort and subgroups.

Odds Ratios of Excessive Birth Weight for BMI Categories			
Birth Weight	Cases/ Controls	OR (95% CI:); *p*	AOR * (95% CI:); *p*
Macrosomia (>4000 g) (*n* = 97) **			
Whole cohort			
Obesity	23/62	4.37 (2.47−7.7); <0.001	3.21 (1.69−6.1); <0.001
Overweight	26/135	2.27 (1.35−3.82); 0.002	1.42 (0.8−2.51); 0.234
Normal BMI	44/518	1	1
Underweight	4/40	1.18 (0.40−3.44); 0.766	1.53 (0.50−4.71); 0.461
‘Healthy’ subgroup			
Obesity	9/26	4.93 (2.12−11.49); <0.001	4.33 (1.71−10.96); 0.002
Overweight	18/96	2.67 (1.42−5.01); 0.002	1.65 (0.82−3.34); 0.164
Normal BMI	29/413	1	1
Underweight	4/30	1.90 (0.63−5.76); 0.257	2.56 (0.79−8.29); 0.117
GWG in the range			
Obesity	6/13	5.37 (1.83−15.7); 0.002	3.94 (1.2−12.95); 0.024
Overweight	4/35	1.33 (0.43−4.14); 0.623	1.06 (0.32−3.52); 0.927
Normal BMI	19/221	1	1
Underweight	3/14	2.49 (0.66−9.44); 0.179	3.43 (0.83−14.26); 0.090
LGA (>90th percentile) (*n* = 99) ***			
Whole cohort			
Obesity	24/64	3.94 (2.26−6.9); <0.001	3.05 (1.65−5.6); <0.001
Overweight	23/137	1.76 (1.04−3.00); 0.037	1.23 (0.7−2.17); 0.478
Normal BMI	48/504	1	1
Underweight	4/36	1.17 (0.40−3.42); 0.779	1.43 (0.47−4.33); 0.531
‘Healthy’ subgroup			
Obesity	8/26	4.19 (1.75−10.04); 0.001	3.66 (1.45−9.28); 0.006
Overweight	16/98	2.22 (1.16−4.23); 0.015	1.49 (0.74−3.01); 0.264
Normal BMI	30/408	1	1
Underweight	4/27	2.02 (0.66−6.14); 0.218	2.50 (0.78−7.99); 0.123
GWG in the range			
Obesity	4/18	2.64 (0.81−8.6); 0.108	1.97 (0.56−6.89); 0.289
Overweight	4/39	1.22 (0.39−3.8); 0.732	0.99 (0.30−3.3); 0.993
Normal BMI	18/214	1	1
Underweight	3/13	2.74 (0.72−10.52); 0.141	3.12 (0.74−13.14); 0.121

* AOR: adjusted odds ratios (with 95% CI: confidence intervals) calculated in the multiple logistic regression (*p*-value < 0.05 was assumed to be significant) and the results were obtained after adjustment for: excessive gestational weight gain (GWG), primiparity, maternal age, maternal height, smoking in the first trimester, fetal sex, preeclampsia, gestational diabetes mellitus in the current pregnancy and gestational age at childbirth (in the subgroups, hypertension and diabetes or GWG were excluded); ** analyses covered cases vs. newborns 2500−4000 g; *** analyses covered cases vs. newborns 10−90th percentile. Subgroup of ‘Healthy’ women: Women who did not develop either diabetes or hypertension in the current pregnancy. LGA: Large-for-gestational age; BMI: Body mass index; GWG: Gestational weight gain.

**Table 4:** The adjusted odds ratios of lower birth weight for pre-pregnancy BMI, in the whole cohort and subgroups.

Odds Ratios of Lower Birth Weight for BMI Categories			
Birth Weight	Cases/ Controls	OR (95% CI:); *p*	AOR * (95% CI:); *p*
LBW (<2500 g) ** (*n* = 60)			
Whole cohort			
Obesity	13/62	3.39 (1.69−6.81); 0.001	1.76 (0.54−5.72); 0.349
Overweight	12/135	1.44 (0.72−2.87); 0.301	1.51 (0.52−4.41); 0.454
Normal BMI	32/518	1	1
Underweight	3/40	1.21 (0.36−4.14); 0.757	0.42 (0.05−3.57); 0.428
‘Healthy’ subgroup			
Obesity	2/26	1.59 (0.35−7.17); 0.547	1.10 (0.16−7.43); 0.925
Overweight	6/96	1.29 (0.51−3.3); 0.594	0.92 (0.21−4.01); 0.914
Normal BMI	20/413	1	1
Underweight	1/30	0.69 (0.09−5.31); 0.720	0.49 (0.05−5.12); 0.547
GWG in the range			
Obesity	6/13	17.00 (4.8−60.1); <0.001	17.42 (1.5−202.6); 0.022
Overweight	8/35	8.42 (2.76−25.7); <0.001	9.07 (1.29−63.70); 0.027
Normal BMI	6/221	1	1
Underweight	3/14	7.89 (1.78−34.93); 0.006	2.51 (0.07−96.7); 0.622
SGA *** (*n* = 56)			
Whole cohort			
Obesity	6/63	1.40 (0.57−3.47); 0.466	0.91 (0.33−2.52); 0.861
Overweight	13/137	1.40 (0.72−2.72); 0.327	1.33 (0.64−2.74); 0.443
Normal BMI	34/500	1	1
Underweight	3/36	1.23 (0.36−4.18); 0.746	1.27 (0.36−4.51); 0.707
‘Healthy’ subgroup			
Obesity	2/26	1.64 (0.36−7.42); 0.521	1.46 (0.31−6.93); 0.638
Overweight	6/98	1.31 (0.51−3.35); 0.580	1.19 (0.43−3.32); 0.738
Normal BMI	19/405	1	1
Underweight	2/27	1.58 (0.35−7.14); 0.553	1.46 (0.31−6.88); 0.630
GWG in the range			
Obesity	2/18	1.81 (0.38−8.66); 0.457	1.75 (0.29−10.5); 0.543
Overweight	4/39	NA	1.28 (0.33−5.03); 0.722
Normal BMI	13/212	1	1
Underweight	1/13	1.25 (0.15−10.34); 0.833	1.89 (0.21−17.1); 0.572

* AOR: adjusted odds ratios (with 95% CI: confidence intervals) calculated in the multiple logistic regression (*p*-value < 0.05 was assumed to be significant) and the results were obtained after adjustment for: Excessive gestational weight gain (GWG), primiparity, maternal age, maternal height, smoking in the first trimester, fetal sex, preeclampsia, and gestational diabetes mellitus in the current pregnancy, and gestational age at childbirth (in the subgroups, hypertension and diabetes or GWG were excluded); ** analyses covered the cases vs. newborns 2500−4000 g; *** analyses covered the cases vs. newborns 10−90th percentile without fetal growth restriction). SGA: small-for-gestational age (birth weight < 10th percentile without fetal growth restriction); ‘Healthy’ subgroup: Women who did not develop either diabetes or hypertension in the current pregnancy; BMI: Body mass index; GWG: Gestational weight gain.

**Table 5:** The adjusted odds ratios of fetal growth restriction (FGR) for pre-pregnancy BMI, in the whole cohort and subgroups.

Odds Ratios of FGR for BMI Categories			
Birth Weight	Cases/ Controls	OR (95% CI:); *p*	AOR * (95% CI:); *p*
FGR (*n* = 21) **			
Whole cohort			
Obesity	5/91	2.64 (0.91−7.7); 0.075	3.12 (1.02−9.54); 0.045
Overweight	0/170	NC	NC
Normal BMI	12/576	1	1
Underweight	4/43	4.47 (1.38−14.4); 0.012	3.84 (1.13−13.0); 0.031
‘Healthy’ subgroup			
Obesity	1/36	1.57 (0.19−12.9); 0.675	2.37 (0.27−20.84); 0.438
Overweight	0/118	NC	NC
Normal BMI	8/452	1	1
Underweight	2/33	3.42 (0.7−16.78); 0.129	2.52 (0.49−12.97); 0.268
GWG in the range			
Obesity	1/24	3.33 (0.33−33.3); 0.305	4.06 (0.38−43.07); 0.245
Overweight	0/47	NC	NC
Normal BMI	3/240	1	1
Underweight	3/17	14.12 (2.65−75.3); 0.002	11.82 (1.95−71.6); 0.007

* AOR: adjusted odds ratios (with 95% CI: confidence intervals) calculated in the multiple logistic regression (*p*-value < 0.05 was assumed to be significant) and the results were obtained after adjustment for: maternal age, primiparity, GWG above the range, and prior pregnancy induced hypertension (GWG were excluded in the subgroup of the GWG in the range); ** the analysis covered mothers with FGR newborns vs. mothers with newborns without FGR. FGR: Fetal growth restriction. ‘Healthy’ subgroup: Women who did not develop either diabetes or hypertension in the current pregnancy; BMI: Body mass index; GWG: Gestational weight gain.

## Data Availability

The data presented in this study are available on request from the corresponding author. The data are not publicly available as it contains a variety of patient information and covers a much wider range than needed for the analyzes presented here.

## Supplementary Materials

The following are available online at https://www.mdpi.com/article/10.3390/nu13041213/s1, Figure S1. The risk profiles of lower birth weight for pre-pregnancy body mass index (BMI) in the whole cohort and subgroup of normal gestational weight gain (GWG), calculated on a sliding window for 30 observations, Table S1. Causal mediation analysis. Mediation effect of excessive gestational weight gain (GWG), Table S2. The adjusted odds ratios of abnormal birth weight for categories of gestational weight gain (GWG) in the subgroup of normal pre-pregnancy BMI.

## Abbreviations

AGA	Appropriate-for-gestational age
AOR	Adjusted odds ratio
APGAR	Appearance, pulse, grimace, activity, respiration
BMI	Body mass index
CI	Confidence intervals
CRL	Crown-rump length
DOHaD	Developmental Origins of Health and Disease
FGR	Fetal growth restriction
GDM	Gestational diabetes mellitus
GWG	Gestational weight gain
IL	Interleukin
IOM	Institute of Medicine
IQR	Interquartile ranges
LBW	Low birth weight
LGA	Large-for-gestational age
MUAC	Mid-upper arm circumference
OGTT	Oral glucose tolerance test
OR	Odds ratio
PE	Preeclampsia
PIH	Pregnancy-induced hypertension
PlGF	Placental growth factor
sFLT	Soluble FMS-like tyrosine kinase
SGA	Small-for-gestational age
TNF-α	Tumor necrosis factor-alpha
WHO	World Health Organization
