# Supplementary material for: Maternal Obesity and Risk of Low Birth Weight, Fetal Growth Restriction, and Macrosomia: Multiple Analyses
Source: Nutrients. 2021 Apr 7;13(4):1213. doi: 10.3390/nu13041213 (PMC8067544; doi:10.3390/nu13041213)
Supplement: Supplementary file 1 [file nutrients-13-01213-s001.zip › Figure S1.docx]

**
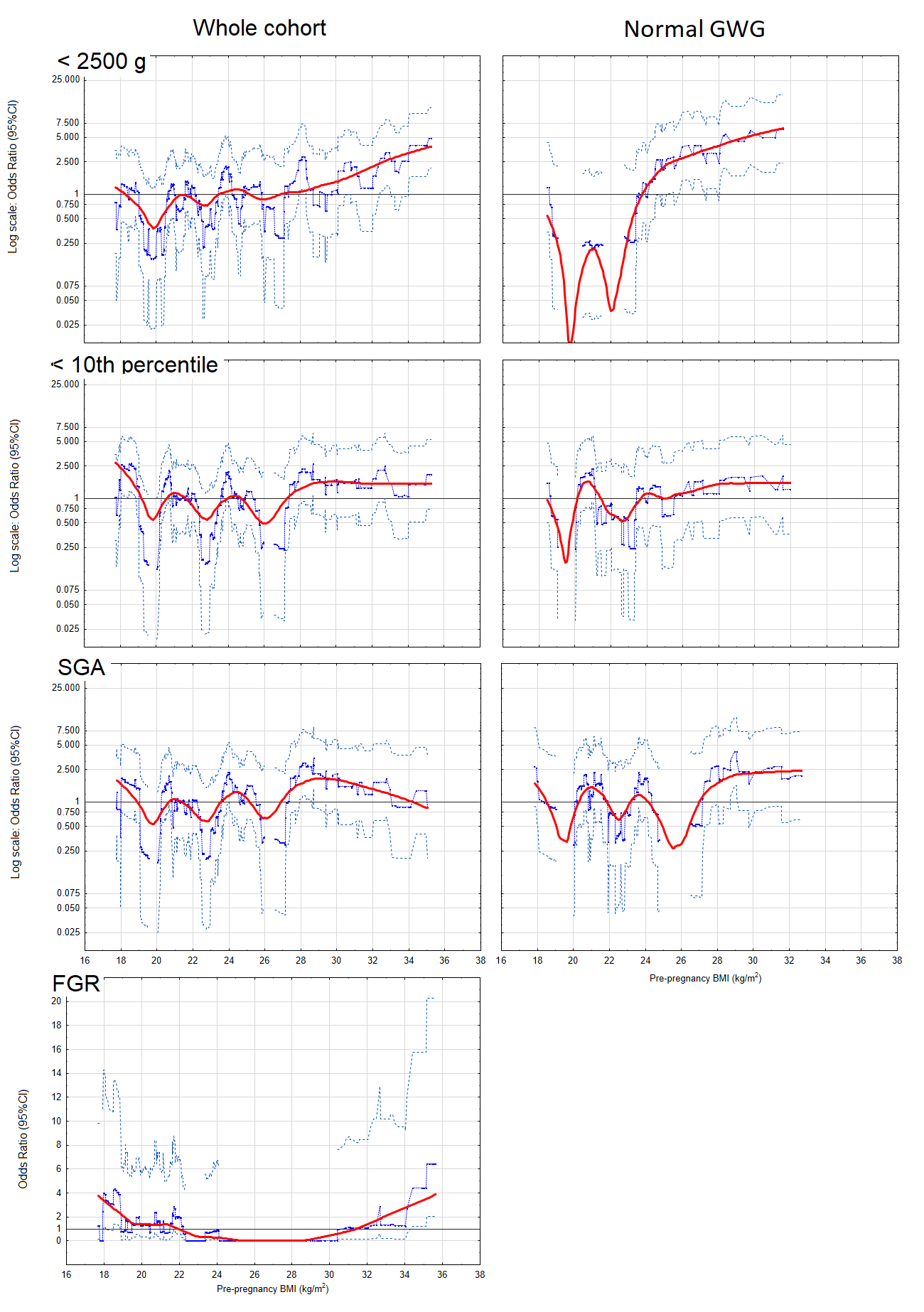
**

**Figure S1.** The risk profiles of lower birth weight for pre-pregnancy body mass index (BMI) in the whole cohort and subgroup of normal gestational weight gain (GWG), calculated on a sliding window for 30 observations. The graphs illustrate the changes in the odds ratios (OR) of the birth weight for the changes in the BMI values (kg/m²). The odds ratios (blue points) were calculated on a sliding window (for 30 observations) and the red curves show the risk profiles (smoothed with the Lowess method). The points above the horizontal line indicate an increased risk, and the points below this line correspond to a reduction in risk. The light blue points represent the upper and lower limits of the 95% confidence intervals (CI). SGA: small-for-gestational age (birth weight < 10th percentile without FGR cases); FGR: fetal growth restriction.
