# Supplementary material for: Maternal Obesity and Risk of Low Birth Weight, Fetal Growth Restriction, and Macrosomia: Multiple Analyses
Source: Nutrients. 2021 Apr 7;13(4):1213. doi: 10.3390/nu13041213 (PMC8067544; doi:10.3390/nu13041213)
Supplement: Supplementary file 1 [file nutrients-13-01213-s001.zip › Table S1.docx]

**Table S1.** Causal Mediation Analysis. Mediation effect of excessive gestational weight gain (GWG).

|  | **Causal mediation effect of excessive GWG,**  **Independent variable: BMI categories,**  **Reference level: Normal BMI** |
| --- | --- |
| **Dependent variable** | **Estimate (95% CI ); p-value** |
| LBW (< 2,500g) |  |
| Whole cohort | -0.01347 (-0.02653 − 0.00); 0.02 * |
| Healthy women | -0.01347 (-0.02653 − 0.00); 0.02 * |
| SGA |  |
| Whole cohort | -0.00300 (-0.01883 − 0.01); 0.56 |
| Healthy women | 0.00194 (-0.01185 − 0.02); 0.78 |
| Macrosomia |  |
| Whole cohort | 0.02625 (0.00748 − 0.05); <0.0001 *** |
| Healthy women | 0.02751 (0.00638 − 0.05); <0.0001 *** |

* / ***Statistical significance levels. LBW: low birth weight; SGA: small-for-gestational age (birth weight < 10th percentile without fetal growth re-striction); Macrosomia: birth weight > 4000g; ‘Healthy’ women: women who did not develop either diabetes or hypertension in the current pregnancy; BMI: body mass index; GWG: gestational weight gain.
