# Supplementary material for: Maternal Obesity and Risk of Low Birth Weight, Fetal Growth Restriction, and Macrosomia: Multiple Analyses
Source: Nutrients. 2021 Apr 7;13(4):1213. doi: 10.3390/nu13041213 (PMC8067544; doi:10.3390/nu13041213)
Supplement: Supplementary file 1 [file nutrients-13-01213-s001.zip › Table S2.docx]

**Table S2.** The adjusted odds ratios of abnormal birth weight for categories of gestational weight gain (GWG) in the subgroup of normal pre-pregnancy BMI.

|  | **Odds ratios of excessive birth weight for GWG categories** | | | |  |  |
| --- | --- | --- | --- | --- | --- | --- |
| **Birth weight** | | **Cases/controls** | **OR (95% CI:); *p*** | **AOR * (95% CI:); *p*** | | |
| **Macrosomia (> 4000 g) ****  **(n = 97)** | |  |  |  | | |
| Normal BMI | |  |  |  | | |
| GWG above the range | | 21/141 | 1.73 (0.9−3.34); 0.100 | 2.16 (1.07−4.35); 0.031 | | |
| GWG in the range | | 19/221 | 1 | 1 | | |
| GWG below the range | | 4/156 | 0.30 (0.1−0.89); 0.031 | 0.28 (0.09−0.94); 0.039 | | |
| **Birth weight >90^th^ percentile *****  **(n = 99)** | |  |  |  | | |
| Normal BMI | |  |  |  | | |
| GWG above the range | | 22/137 | 1.91 (0.99−3.69); 0.054 | 2.35 (1.18−4.67); 0.015 | | |
| GWG in the range | | 18/214 | 1 | 1 | | |
| GWG below the range | | 8/153 | 0.62 (0.26−1.47); 0.278 | 0.49 (0.2−1.23); 0.128 | | |
| **LBW ( < 2500 g) ****  **(n = 60)** | |  |  |  | | |
| Normal BMI | |  |  |  | | |
| GWG above the range | | 8/141 | 2.09 (0.71−6.15); 0.181 | 1.39 (0.31−6.10); 0.667 | | |
| GWG in the range | | 6/221 | 1 | 1 | | |
| GWG below the range | | 18/156 | 4.25 (1.65−10.95); 0.003 | 3.71 (1.1−12.55); 0.035 | | |
| **SGA *****  **(n = 56)** | |  |  |  | | |
| Normal BMI | |  |  |  | | |
| GWG above the range | | 11/136 | 1.32 (0.57−3.03); 0.514 | 1.15 (0.47−2.79); 0.758 | | |
| GWG in the range | | 13/212 | 1 | 1 | | |
| GWG below the range | | 10/152 | 1.07 (0.46−2.51); 0.871 | 0.90 (0.36−2.23); 0.818 | | |
| **FGR**  **(n = 21)** | |  |  |  | | |
| Normal BMI | |  |  |  | | |
| GWG above the range | | 1/168 | 0.48 (0.05−4.62); 0.522 | 0.42 (0.04−4.2); 0.458 | | |
| GWG in the range | | 3/240 | 1 | 1 | | |
| GWG below the range | | 8/168 | 3.81 (1.0−14.57); 0.051 | 3.74 (0.97−14.48); 0.056 | | |

* AOR: adjusted odds ratios calculated in the multiple logistic regression (with 95% CI: confidence intervals) and p-value < 0.05 was assumed to be significant: the results were adjusted for pre-pregnancy BMI, primiparity, maternal age, maternal height, smoking in the first trimester, fetal sex, preeclampsia and gestational diabetes in the current pregnancy and gestational age at childbirth (for birth weight in grams or percentiles); the odds ratios of FGR were adjusted for pre-pregnancy BMI, primiparity, maternal age, and prior hypertension; ** analyses covered cases vs birth weight 2500-4000 g; *** analyses covered cases vs birth weight 10-90th percentile. LBW: low birth weight; SGA: small-for-gestational age (birth weight < 10th percentile without FGR cases); FGR: fetal growth restriction.
